# NDM-1 plasmid clustering reflects clonal transmission of Klebsiella pneumoniae ST147 in four hospitals in Berlin, Germany

**DOI:** 10.1186/s13756-025-01639-x

**Published:** 2025-10-02

**Authors:** Anna Weber, Luisa Denkel, Christine Geffers, Axel Kola, Friederike Maechler

**Affiliations:** Institute of Hygiene and Environmental Medicine, Charité – Universitätsmedizin Berlin, Berlin, Germany

**Keywords:** Klebsiella pneumoniae, NDM-1, Hospital outbreak, Plasmids, Germany

## Abstract

**Background:**

In recent years, the detection of *Klebsiella pneumoniae* (KLPN) producing New Delhi metallo-β-lactamase (NDM), particularly NDM-1, has increased in Germany. Plasmids play a crucial role in the dissemination of NDM-1, facilitating its persistence in both clinical and environmental reservoirs. Between 2021 and 2024, a substantial number of NDM-1-producing KLPN isolates were detected across multiple hospital sites in Berlin. This study aimed to investigate a potential multi-site outbreak involving NDM-1-producing KLPN, and to assess the role of clonal versus plasmid-mediated dissemination.

**Methods:**

We performed short-read sequencing for all isolates, complemented by long-read sequencing for a subset (Illumina and Oxford Nanopore Technologies). Core genome multi locus sequence typing (cgMLST) was conducted using SeqSphere+. NDM-1 plasmids were characterized with the MOB-suite tools. Reference plasmids were reconstructed from hybrid assemblies using TaDReP. Short-read sequences from all isolates were aligned against these reference plasmids to assess genetic relatedness.

**Results:**

Analysis of 57 NDM-1 plasmid carrying KLPN isolates at the clonal level revealed three distinct outbreak clusters (O1-O3). These corresponded to three unique NDM-1 reference plasmids: p1 (54.0 kb IncFIB(pQil)), p2 (54.3 kb IncR), and p3 (355.5 kb; no Inc type specified). Plasmid clustering from short-reads was consistent with the clonal clusters.

**Conclusions:**

Both plasmid-level analysis and cgMLST yielded congruent results, effectively ruling out the possibility of multi-site, plasmid-mediated NDM-1 transmission. The detection of a globally disseminated NDM-1 plasmid within one of the clonal clusters highlights its potential role in the spread of resistance during the recent surge of NDM-1 carrying KLPN isolates in Germany.

**Supplementary Information:**

The online version contains supplementary material available at 10.1186/s13756-025-01639-x.

## Background

*Klebsiella pneumoniae* (KLPN) is a Gram-negative opportunistic pathogen that causes severe infections, particularly in healthcare settings. Its increasingly multidrug resistance poses a significant public health concern worldwide [1]. One of the most critical resistance mechanisms in KLPN is the production of carbapenemases, enzymes that hydrolyze carbapenems, which are often considered last-resort antibiotics [2]. Among the various carbapenemases, New Delhi metallo-β-lactamase (NDM) has gained particular attention due to its increased detection. In Europe, and especially in Germany, the incidence of NDM-1-producing KLPN has risen considerably in recent years, as reflected by data from the National Reference Centre for Multidrug-Resistant Gram-negative Bacteria, which show a marked increase in carbapenemase-producing isolates, including NDM-1, between 2012 and 2021 [1]. Furthermore, Sandfort et al. reported that NDM-1 plays a major role in the spread of carbapenem resistance in Germany [3]. NDM resistance is primarily disseminated through plasmid-mediated horizontal gene transfer, enabling its rapid spread within and across different bacterial strains and species. Mobile genetic elements, such as conjugative plasmids, facilitate the transmission of NDM-1, contributing to its persistence in both clinical and environmental settings. The ability of these plasmids to transfer between bacterial hosts enhances the complexity of controlling outbreaks and underscores the importance of characterizing plasmid diversity in surveillance efforts [4, 5]. Research on plasmid-associated outbreaks has intensified in the past few years [6, 7, 8].

Since 2021, we observed a rising number of outbreaks with NDM-1-producing KLPN across several hospital sites at Charité – Universitätsmedizin Berlin. This study investigates whether these outbreaks were part of a single, plasmid-associated transmission cluster spanning multiple hospital sites.

## Methods

### Study design

The study was conducted at Charité – Universitätsmedizin Berlin, a large acute care facility with four hospital sites across three districts of Berlin. The isolates were identified retrospectively through a search of the patient data management system for all NDM positive samples collected between January 1, 2021 and June 18, 2024. A total of 251 isolates were retrieved, of which 71% (178) were identified as KLPN, 17% (43) as *Escherichia coli*, 14% [34] as *Acinetobacter baumannii complex* and 6% [14] as *Citrobacter freundii* (all other species accounted for less than 4%). Given the occurrence of several apparently independent clonal outbreaks of NDM-1-producing KLPN, the investigation was focused on this species. Further selection criteria included availability of sequencing data and confirmation of an NDM-1-carbapenemase production, resulting in 57 KLPN included in the subsequent analyses. These isolates originated from both clinical and screening samples, as well as environmental sources, and were sequenced as part of routine hospital surveillance and transmission investigations.

### Whole genome sequencing

Genomic DNA was extracted from overnight cultures on blood agar plates using the DNeasy Ultra Clean kit (QIAGEN, Hilden, Germany) according to the manufacturer’s instructions. Short-read sequencing libraries were prepared using the Nextera XT Kit (Illumina Inc., San Diego, USA), and sequenced on the MiSeq platform (Illumina Inc., San Diego, USA). Representing the various outbreak clusters, a subset of isolates (*n* = 4) underwent long-read sequencing on the MinION platform (Oxford Nanopore Technologies Ltd., Oxford, United Kingdom). Library preparation for long-read sequencing was carried out using the Native Barcoding Kit, and sequencing was conducted on R10.4 flow cells.

### Bioinformatic analyses

All isolates were sequenced using short-read (Illumina). *De novo* assemblies were generated with SeqSphere + software (Ridom, version 9.0.10) [9] using the integrated SKESA assembler (version 2.3.0) under default settings. Long-read sequencing data (Oxford nanopore Technologies) were quality-trimmed with Rasusa (version 2.0.0) to optimize read length and coverage [10]. Hybrid assemblies were generated with Flye (version 2.9.1), followed by polishing with short-read data using polypolish (version 0.6.0) and pypolca (version 0.3.1) [11, 12, 13, 14]. Alternatively, genomes were assembled combining both short- and long-read data with Unicycler (version 0.5.0) [15].

Following assembly, all isolates underwent core genome multilocus sequence typing (cgMLST) within SeqSphere+, based on the published cgMLST task template scheme for *Klebsiella pneumoniae* at default settings (reference genome: NC_012731.1; allelic distance threshold: 15). FASTA contigs from *de novo* assemblies were analyzed with Kleborate (version 3.1.2) to determine virulence and resistance profiles, providing further insight into strain characteristics [16].

The frequent occurrence of NDM-1 KLPN prompted further investigations into the characteristics of NDM-1 plasmids. Only plasmids carrying the bla_NDM−1_ gene were retained for downstream analysis; all others were excluded). NDM-1-harboring plasmids were characterized with the MOB-suite (version 3.1.8) and NCBI AMRFinderPlus (version 3.11.26) [17, 18].

For hybrid assemblies, TaDReP (Targeted Detection and Reconstruction of Plasmids, version 0.9.2) was used to define NDM-1 reference plasmids, which served as alignment targets for short-read data from all isolates (https://github.com/oschwengers/tadrep). TaDReP is a command-line tool designed for targeted identification and clustering of plasmid sequences using draft genome assemblies. It identifies and reconstructs putative reference plasmids from draft assemblies by aligning contigs using BLAST+, applying stringent thresholds for sequence coverage and identity. Only contigs that meet these strict criteria are retained, ensuring that final plasmid reconstructions are based on high-confidence alignments. In the first step, plasmid contigs from hybrid-assembled NDM-1-positive isolates were extracted, characterized, and compared to determine whether they (i) formed a unified NDM-1 plasmid cluster, indicative of a multi-site NDM-1 plasmid outbreak, or (ii) were distinct entities. For this reconstruction of reference plasmids, a minimum plasmid sequence identity of 90% and maximum sequence length difference of 1,800 bp were used. In the second step, the reconstructed reference plasmids from step one were used as alignment targets for all NDM-1 short-read assemblies to assess whether these reference plasmids could be detected in the remaining isolates. Default settings were applied, with a minimum plasmid coverage of 80% and minimum plasmid identity of 90% for plasmid detection in short-reads.

A plasmid cluster was defined as a group of at least two NDM-1-encoding plasmids grouped by similarity and the specific clustering parameters applied within the respective analysis tools.

To construct a maximum-likelihood phylogenetic tree, a core genome SNP alignment was first generated using Snippy (version 4.6.0) [19], with the hybrid-assembled M012837 serving as reference genome. The resulting alignment was then used to infer the phylogeny using FastTree (version 2.1.11) [20, 21] and the tree was visualized with iTOL (version 7) [22, 23]. Reference plasmids were queried against the NCBI nucleotide database (blastn, version 2.15.0) [24], visualized as circular maps with BRIG (version 0.95), and structural variation was further explored through gene cluster alignments generated in clinker (version 0.0.29-1) [25, 26]. All command-line operations are provided as Supplementary material (Supplementary Text S1).

## Results

### Overview of isolates carrying NDM-1

**Fig. 1:**
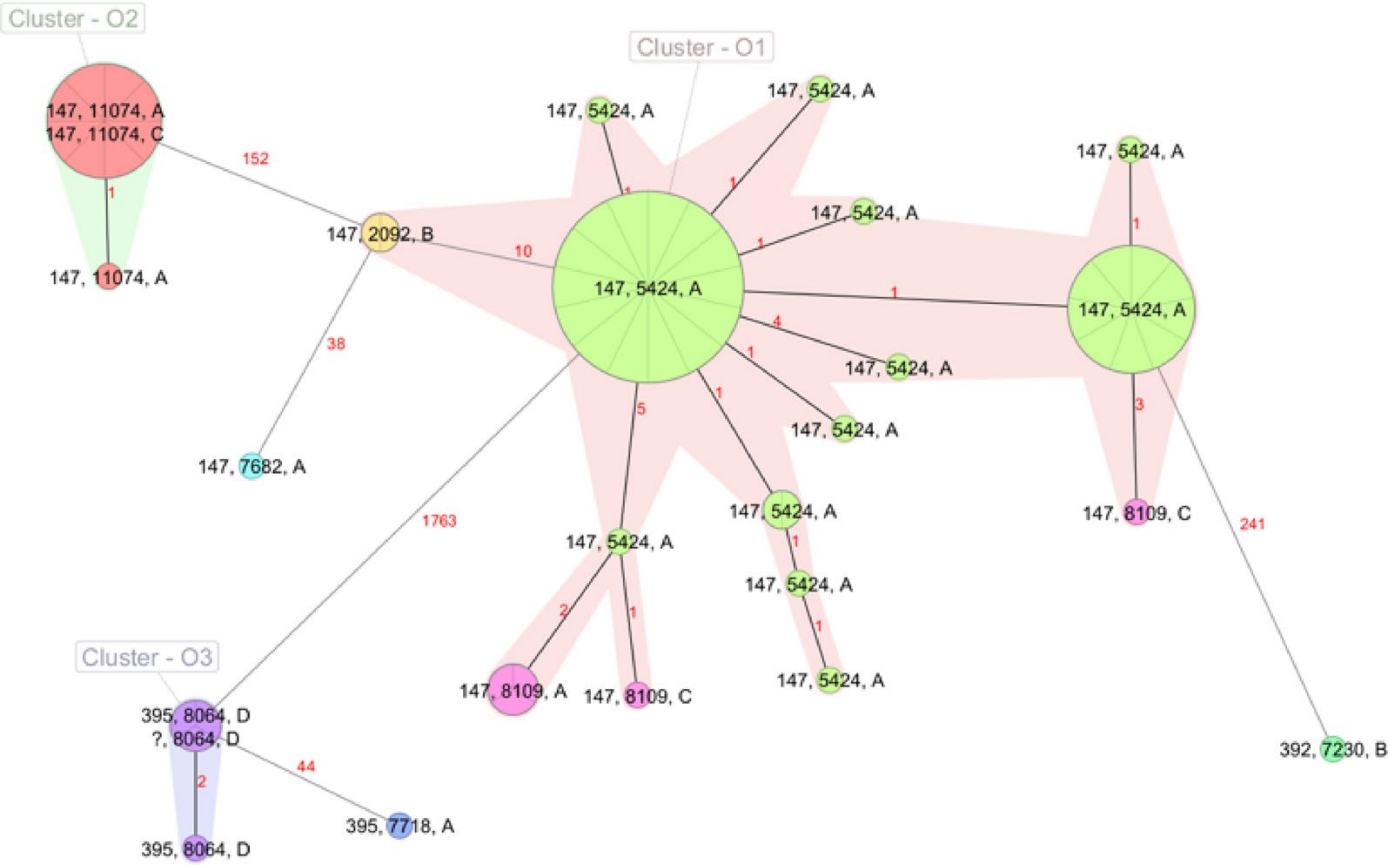
Minimum Spanning tree of all 57 *Klebsiella pneumoniae* isolates based on cgMLST (SeqSphere+) showing sequence type, complex type, and hospital site A-D (cluster distance threshold: 15; “?”: no ST assignment for this sample)

cgMLST identified three outbreak clusters, as shown in Fig. 1. The largest cluster, hereafter referred to as outbreak cluster 1 (O1), comprised 41 isolates collected across three hospital sites (A, B and C). The two smaller clusters, outbreak cluster 2 (O2) and 3 (O3), consisted of 9 and 4 isolates respectively, originating from hospital sites A and C (O2), and D (O3). Detailed information, including year of collection, sampling site and sequence type, is summarized in Table 1, with isolates grouped according to outbreak cluster (O1-O3). To further resolve the genetic relationships among isolates, a core genome SNP-based phylogenetic tree was generated (Figure S1), confirming the clustering patterns observed in the cgMLST analysis. Kleborate analysis revealed distinct virulence profiles among the three groups of clustered isolates (Table 1 and Supplementary Table S1). The Kleborate virulence score summarizes the presence of major virulence loci, including the siderophores yersiniabactin (ybt) and aerobactin (iuc), as well as the genotoxin colibactin (clb). The score ranges from 0 to 5 (0 = none detected; 1 = yersiniabactin (ybt) only; 2 = colibactin (clb); 3 = aerobactin (iuc) only; 4 = both aerobactin and yersiniabactin, but not colibactin; 5 = all three loci present (ybt, clb, and iuc)) [16].

Of all isolates, 80% were classified as hospital-acquired. Each of the three clusters O1-O3 represented a distinct outbreak investigated through routine surveillance and outbreak response activities.

The outbreak corresponding to cluster O1 occurred at hospital A, primarily affecting a hematological / oncological unit. O1 strains were also detected in environmental samples, and the outbreak has remained ongoing for over four years (2021–2024). Similarly, cluster O2 was linked to an outbreak in hospital A, specifically involving the internal medicine intensive care and nephrology units, with cases emerging in late 2023 and continuing into 2024. In contrast, cluster O3 was associated with an outbreak in hospital D, affecting the cardiology center, and was confined to the year 2023. Clusters O1 and O2 included a few isolates from multiple hospital sites that were not epidemiologically linked to other isolates within the same outbreak cluster (see epidemiological information in Table S1).

**Table 1:** Characteristics of all *Klebsiella pneumoniae* study isolates (*n* = 57) grouped by outbreak cluster (O1-O3)

Parameter	Outbreak cluster 1 (O1)	Outbreak cluster 2 (O2)	Outbreak cluster 3 (O3)	Singletons
Number of isolates	41	9	4	3
ST and CT	ST147 CT5424/8109/2092	ST147 CT11074	ST395 CT8064	ST395 CT7718/7230, ST147 CT7682
Hospital	A, B and C	A and C	D	A and B
Collection year, n (%)	2021, *n* = 9 (22%) 2022, *n* = 19 (46%) 2023, *n* = 11 (27%) 2024, *n* = 2 (5%)	2023, *n* = 2 (22%) 2024, *n* = 7 (78%)	2023, *n* = 3 (75%) 2024, *n* = 1 (25%)	2022, *n* = 2 (66%) 2024, *n* = 1 (33%)
Sampling site, n (%)	Rectal swabs, *n* = 29 (70%) Blood culture, *n* = 3 (7%) Urine, *n* = 2 (5%) Environmental, *n* = 2 (5%) Other, *n* = 5 (12%)	Rectal swabs, *n* = 1 (11%) Nasal throat swabs, *n* = 4 (44%) Respiratory tract secretions, *n* = 3 (33%) Other, *n* = 1 (11%)	Wound swab, *n* = 1 (25%) Intraoperative swab, *n* = 1 (25%) Not reported, *n* = 2 (50%)	Rectal swabs, *n* = 1 (33%) Urine, *n* = 1 (33%) Intraoperative swab, *n* = 1 (33%)
Kleborate virulence score value*, n (%)	Score 1, *n* = 41 (100%)	Score 3, *n* = 9 (100%)	Score 4, *n* = 4 (100%)	Score 1, *n* = 1 (33%) Score 4, *n* = 2 (66%)
Virulence factors and characteristics (Kleborate), n (%)	• Yersiniabactin, *n* = 41 (100%) • KL64, *n* = 41 (100%)	Aerobactin, *n* = 9 (100%) rmpA, *n* = 9 (100%) KL35, *n* = 9 (100%)	Yersiniabactin *n* = 4 (100%) Aerobactin, *n* = 4 (100%) rmpA, *n* = 3 (75%) KL39 *n* = 3 (75%), KL2 *n* = 1 (25%)	Yersiniabactin, *n* = 3 (100%) Aerobactin, *n* = 2 (66%) rmpA, *n* = 1 (33%) KL2 *n* = 1 (33%), KL64 *n* = 1 (33%), KL27 *n* = 1 (33%)

*Kleborate virulence score: 0 , none detected; 1 , yersiniabactin (ybt) only; 2 , colibactin (clb) without aerobactin; 3, aerobactin (iuc) only; 4 , both aerobactin and yersiniabactin, but not colibactin; 5 , all three loci present (ybt, clb, and iuc)

### Characterization and alignment of NDM-1-encoding plasmids

Representative isolates from each outbreak cluster (O1–O3) were selected for long-read sequencing based on sequencing quality and metadata (O1: M009157, M012837; O2: M013447; O3: M013506). Two representatives were chosen for O1 due to the large number of associated isolates and the absence of a clear epidemiological link among some of them. TaDReP analysis identified three distinct NDM-1 plasmids, designated as NDM-1 reference plasmids and named according to their associated outbreak clusters: Reference plasmid p1 (representatives of O1: M009157 and M012837), p2 (O2: M013447) and p3 (M013506). As input for reference plasmid reconstruction, all four hybrid assemblies were included. Since each isolate carried only one NDM-1 plasmid, a maximum of four reference plasmids was theoretically possible. TaDReP analysis yielded three distinct reference plasmids (p1–p3), as plasmids from outbreak cluster O1 were identical and merged into a single reference plasmid.

**Fig. 2:**
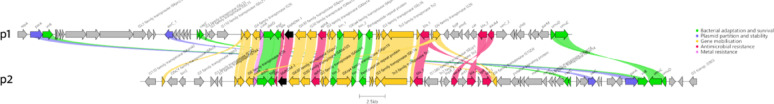
Visualization of NDM-1 reference plasmid p1 and p2. Color-coded blocks indicate conserved regions, connecting lines illustrate homologues sequences and structural rearrangements. An external reference was not used, as the complete plasmids generated here serve as context-specific comparators

The reference plasmid p1 is a 54.0 kb plasmid of incompatibility group (Inc) FIB(pQil). Reference plasmid p2 is a 54.3 kb IncR plasmid, while p3 is a 355.5 kb plasmid with no specified incompatibility group. Detailed characteristics of the reconstructed reference plasmids p1-p3 are provided in Supplementary Table S5. A comparative analysis of the three NDM-1 reference plasmids, highlighting their similarities and differences, is presented in the Supplementary Figure S2. A detailed comparison of p1 and p2 revealed a high degree of synteny across the central region of both plasmids, with multiple conserved genes including genes involved in replication, stability, metal and antimicrobial resistance as shown in Fig. 2. Due to its considerably larger size, plasmid p3 was excluded from Fig. 2; plasmids p1 and p2 were selected based on size similarity and structural comparability. However, notable structural variations were observed, including differences in the presence of multiple insertion sequences (IS elements), absence of specific genes (e.g. repA, folP, emrE, cat - all present in p1, whereas p2 lacks the replication gene), as well as variations in gene order and orientation.

**Fig. 3:**
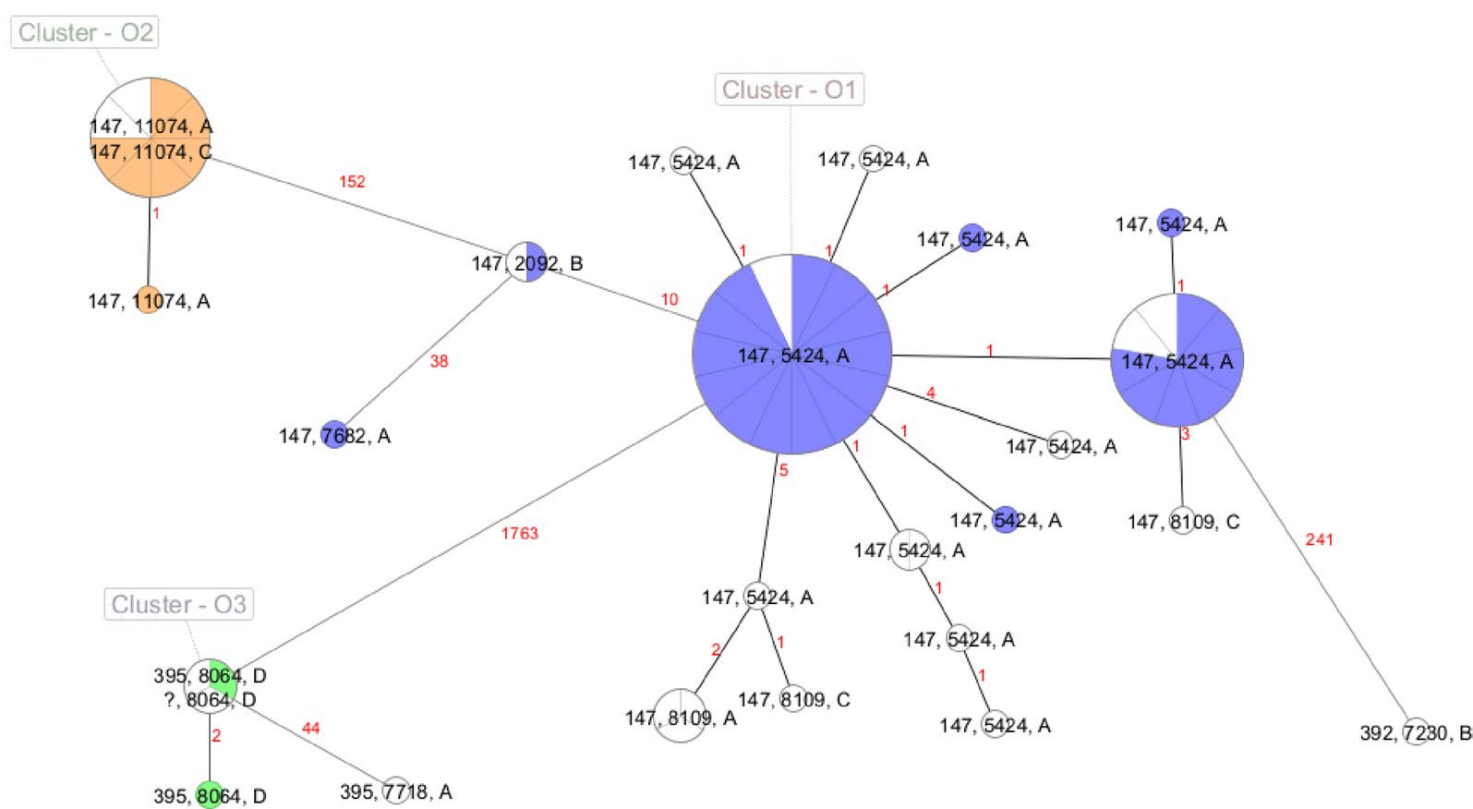
Minimum spanning tree of 57 *Klebsiella pneumoniae* isolates based on cgMLST (SeqSphere+). Nodes are color-coded to indicate the detected NDM-1 reference plasmid as determined by TaDReP alignment results (blue: p1; orange: p2; green p3; white: no reference plasmid assigned)

All short-read isolates not chosen for additional long read sequencing (*n* = 53) were aligned against the reconstructed NDM-1 reference plasmids. The analysis revealed that reference plasmid p1 was detected in 47% (25/53) of the isolates, p2 in 13% (7/53), and p3 in 4% (2/53). Overall, these three NDM-1 reference plasmids accounted for 64% (34/53) of all NDM-1 plasmids identified in this study, with sequence coverages of 80.4%-95.6% and nucleotide identities of 99.7%-100% to the respective reference sequences. Nearly all plasmids in which p1 was detected originated from isolates within outbreak cluster O1 (24/25), with a single plasmid classified as a singleton and not associated with any of the outbreak clusters O1-O3 (Fig. 3). Similarly, all p2-plasmids were linked to isolates from outbreak cluster O2, and all p3-plasmids corresponded to isolates from outbreak cluster O3. The sizes of p1-plasmids ranged from 45.4 to 51.7 kb, as determined by MOB-suite analysis (Table 2), while the reference plasmid p1 had a size of 54 kb. Similarly, all p3-detected plasmids ranged between 351.8 and 357.1 kb in size, with the reference plasmid p3 measuring 355 kb.

The p2-plasmids exhibited sizes between 316.4 and 351.2 kb, with one outlier at 11.8 kb. These findings differ from reference plasmid p2 size of 54.3 kb as determined by TaDReP. During the reconstruction of reference plasmids using TaDReP, two possible plasmids were identified for the O2 representative M013447: one measuring 54.3 kb and another at 296.8 kb. The larger plasmid did not contain any detectable bla_NDM−1_ genes and was thus excluded from further analysis. The large size variation among the p2-aligned plasmids (ranging from 11.8 kb to 323.2 kb) reflects the modular architecture of these functionalized, multi-replicon plasmids and shows the limitation of p2 a reference plasmid. Larger assemblies likely represent full-length plasmids containing additional replicons.

Overall, 36% (*n* = 19) of the NDM-1 plasmids did not correspond to any of the three NDM-1 reference plasmids. Among these, a comparative analysis revealed the presence of both unique singleton NDM-1 plasmids as well as a potential additional plasmid cluster with *n* = 9 plasmids. These clustered plasmids were characterized by a uniform size of 16.8 kb, lacked a specified Inc type, and were all associated with isolates from outbreak cluster O1, as detailed in Table 2.

### Comparative analysis of NDM-1 reference plasmids with NCBI BLAST

A BLAST search for the three reference plasmids against the NCBI nucleotide database revealed several high-confidence matches for the NDM-1 plasmid p1, with coverage and sequence identity exceeding 99% (Supplementary Table S3). These results include a total of eight KLPN isolates collected between 2019 and 2024 from multiple countries, including Russia (CP125103.1), Italy (CP098430.1; CP072928.1), the United States (CP074090.1), Japan (LC521845.1; AP018834.1), Switzerland (CP082992.1), and Croatia (OZ111522.1).

In contrast to the findings for plasmid p1, no matches with an alignment length exceeding 65% were found for plasmids p2 and p3 (Supplementary Table S4 and Table S5).

**Table 2:** Characteristics of NDM-1-encoding plasmids (*n* = 53) aligned with reference plasmids p1-p3

Parameter	p1	p2	p3	No reference plasmid assigned
Number of aligned NDM-1 plasmids	25	7	2	19
Outbreak cluster assignment at core genome level, (n)	O1 (24) Singletons (1)	O2 (7)	O3 (2)	O1 (15) O2 (1) O3 (1) Singletons (2)
Plasmid size, kb (n)	45.4–51.7 kb (25)	11.8 kb (1) 316.4–323.2 kb (6)	351.8–357.1 kb (2)	16.8 kb (9) 33.1 kb (1) 46.0-50.5 kb (5) 120.4 kb (1) 212.9–215.8 kb (2) 307.7 kb (1)
Secondary cluster ID, (n)	AH565 (25)	AI436 (6) AE581 (1)	AI436 (2)	AL454 (9) AH565 (5) AI436 (2) AL185 (1) AI077 (1) AB189 (1)
Inc group, Name (n)	IncFIB (25)	IncFIB: IncHI1B: IncR: rep_cluster_1254 (6) - (1)	IncFIB: IncHI1B: IncR: rep_cluster_1254 (2)	- (9) IncFIB (6) IncFIB: IncFII: rep_cluster_2183 (1) IncFIB: IncHI1B (1) IncR (1) IncFIB: IncHI1B: IncR: rep_cluster_1254 (1)
AMRFinderPlus targets, Name (n)	blaCTX-M-15 (18) blaNDM-1 (25)	blaCTX-M-15 (6) blaNDM-1 (7)	blaCTX-M-15 (2)	blaCTX-M-15 (15) blaOXA-48 (1)

## Discussion

This study investigated a potential NDM-1 plasmid outbreak across multiple hospital sites by analyzing 57 KLPN isolates at both clonal and plasmid levels. Our findings indicated that no widespread NDM-1 plasmid outbreak occurred, as we identified three distinct NDM-1 plasmids (p1, p2, p3), each corresponding to separate outbreak clusters O1 - O3 based on cgMLST. Thus, the spread of NDM-1 was associated with clonal dissemination of distinct NDM-1-encoding plasmids, rather than by a single, shared plasmid. Plasmid p1 appears to be globally distributed, as it shows high sequence similarity to published plasmid sequences available in the NCBI database. Consequently, spread of NDM-1 KLPN seems to be driven by clonal transmission and plasmid-mediated dissemination.

The identification of three distinct outbreak clusters (O1, O2, and O3) at the core genome level highlights the clonal diversity of NDM-1-producing KLPN isolates at Charité - Universitätsmedizin Berlin. The isolates from outbreak clusters O1 and O2 correspond to ST147, a common sequence type in Germany. This finding aligns with previous research indicating the widespread presence of ST147 among carbapenemase-producing KLPN in this country between 2008 and 2014 [27]. Moreover, the increase in NDM-1-carbapenemases in Germany between 2016 and 2022 has been attributed to KLPN ST147 [3]. ST147 is a globally disseminated high-risk clone of KLPN, associated with various antimicrobial resistance determinants, including carbapenemases such as NDM-1, OXA-48, and KPC. In Europe, ST147 has been implicated in numerous nosocomial outbreaks, particularly in countries like Italy, Greece, and Spain [28]. In Poland, genomic analyses have demonstrated the continuous spread of NDM-1-producing ST147 strains since 2015, particularly in the Warsaw area [29]. Several studies also reported an increased incidence of ST147 NDM-1 KLPN among patients linked to the war in Ukraine [30, 31, 32]. These findings highlight the role of ST147 as a significant vector for the dissemination of NDM-1 resistance gene, and suggest that cross-border transmission remains a relevant factor in the spread of high-risk clones such as ST147, including a potential importation of ST147 strains into the Charité. ST395, the sequence type of O3 isolates, has also been documented in the literature in relation to the spread of NDM-1 [3].

The Kleborate analysis in this study identified virulent NDM-1 ST147 strains (O2) and hypervirulent NDM-1 ST395 strains. Hypervirulence lacks a universally accepted definition. Various criteria have been proposed, but all are associated with increased pathogenic potential. A recent study suggested that the presence of ≥ 4 of the five biomarkers iucA, iroB, peg-344, rmpA, and rmpA2 is highly sensitive for identifying hypervirulent KLPN strains [33]. In our study, virulence was assessed using the Kleborate virulence score, which assigns the highest score when yersiniabactin, aerobactin, and colibactin loci are all present [16]. Several studies have reported KLPN isolates exhibiting both colistin and carbapenem resistance, along with hypervirulence [34, 35]. In our analyzed strains, no colistin resistance genes (mcr) were detected. Recent studies have recognized KLPN ST147 as a globally distributed, high-risk convergent clone associated with both multidrug resistance and increased virulence. Notably, Turton et al. reported that a high proportion of ST147 isolates (~ 47%) carried IncFIB(pNDM-Mar)/IncHI1B(pNDM-MAR) hybrid plasmids integrating both resistance and virulence determinants [36]. This genetic configuration enhances the pathogenic potential of these strains and poses an increased risk to patients. The coexistence of resistance and virulence genes within single plasmids facilitates the rapid dissemination of these traits across bacterial populations, complicating treatment options and infection control measures [36, 37]. In our study, we did not identify any NDM-1 plasmids carrying both resistance and virulence genes.

Using long-read sequencing (hybrid assemblies) we identified three NDM-1 reference plasmids (p1, p2 and p3) with different characteristics as already described, each forming its own plasmid cluster. Thereby, the plasmid analysis identified clone-specific NDM-1 plasmids that closely matched with the cgMLST outbreak clusters O1-O3. According to MOB-suite analysis of short-read data, variations in plasmid size and genetic content were consistent with the outbreak cluster classification and also corroborates that the presence of NDM-1 was not driven by a single uniform NDM-1 plasmid, but rather associated with clonal dissemination of multiple, distinct NDM-1-encoding plasmids. Notably, there was no overlap in plasmid assignment between different outbreak clusters, further supporting the notion that antimicrobial resistance dissemination in this setting was primarily driven by clonal transmission, with limited evidence for horizontal plasmid transfer between unrelated strains. In this study, plasmid clustering largely mirrored clonal grouping, consistent with findings from Akintayo et al., who reported a complex picture of clonal transmission and plasmid exchange [35]. Plasmid-mediated transmission of resistance and/or virulence genes, alongside clonal dissemination, may further complicate outbreak management. However, some outbreaks appear to be predominantly plasmid-mediated, as observed in the work by Schweizer et al. [38], where the plasmid was transferred across multiple bacterial species, facilitating the spread of resistance.

Despite the promising insights gained from our plasmid analysis, a comparison of MOB-suite results and TaDReP alignments revealed discrepancies in the size of the NDM-1 reference plasmid p2 and the corresponding aligned plasmids identified from short-read assemblies. This suggests that the grouping of short-read contigs containing similar plasmid partial sequences and subsequent processing of these FASTA files may result in chimeric assemblies comprising sequences from multiple, closely related plasmids. Accordingly, contigs from secondary cluster IDs assigned in MOB-Suite, like AI436 in outbreak cluster O2 isolates, could in fact encompass multiple plasmids with homologous sequences. Thus, fragmented genomes and structurally similar plasmids complicate the analysis. Using TaDReP, 64% of the NDM-1 plasmids were assigned to one of the three reference plasmids, with p1 representing the largest proportion at 47%. The 36% of NDM-1 plasmids that could not be assigned to p1-p3 reflect the diversity of the NDM-1 plasmids in this study population. Some of the unassigned plasmids could represent a distinct NDM-1 plasmid cluster within O1 (plasmid size 16.8 kb, no specified Inc type). Alternatively, due to their structural similarity to p1, they could merely be fragments of p1, potentially resulting from structural rearrangements, recombination or incomplete plasmid assembly.

The BLAST analysis of the NDM-1 reference plasmids (p1-p3) in the NCBI database revealed that plasmid p1 has already been described in association with the occurrence of NDM-1-producing *Klebsiella pneumoniae* strains from various countries. An example is the study by Akintayo et al., which investigated clonal and plasmid-mediated transmission in colistin- and carbapenem-resistant *Klebsiella pneumoniae* [35]. The ST147 isolate Zagreb045 (OZ111522.1), identified in Croatia in 2024, harbors a 54 kb IncFIB(pQIL) plasmid carrying the bla_NDM−1_ gene. While the study generally discussed potential plasmid transmissions, it did not provide specific insights into this particular plasmid. We did not identify clonal-level matches between this isolate and those containing the p1-plasmid among our own study isolates (data not shown). In summary, p1 appears to be a geographically widespread plasmid; however, to date, it has been documented exclusively in KLPN and not in other bacterial species.

This study has several limitations. Only KLPN isolates were examined, while other potential reservoir species were not part of the analysis. Since other species may also contribute to the dissemination of NDM-1, a broader investigation would be necessary to determine the full extent of NDM-1 plasmid distribution. Moreover, our study is limited by the small number of isolates subjected to long-read sequencing. The reference plasmids were reconstructed from four hybrid assemblies, capturing only 64% of all NDM-1 plasmids identified. The remaining plasmids were not represented by any of the reference plasmids. Another limitation lies in the retrospective nature of the study, which focused on previously sequenced NDM-positive KLPN isolates collected during routine admission surveillance and outbreak investigations. These isolates were not systematically sampled across a defined time period, limiting the ability to draw broader conclusions about the epidemiology of clonal transmission and horizontal plasmid transfer. Despite the detailed characterization, the plasmid analysis did not provide a clear explanation for the presence of similar plasmids and isolates across multiple hospital sites, as no direct epidemiological link could be established.

A strength of this study lies in its combined analysis of clonal transmission and plasmid dissemination among more than 50 NDM-1-producing KLPN isolates based on short-read and selected long-read sequencing data. Although the study was retrospective and based on isolates collected during routine hospital surveillance and outbreak investigations over a period of 4.5 years, the integration of molecular typing with epidemiological data, allowed for a refined understanding of the transmission dynamics in outbreak setting.

To sum up, our plasmid analysis effectively ruled out a multi-site NDM-1 plasmid outbreak across the four different hospital sites studied. Both cgMLST and plasmid analysis yielded congruent results, forming consistent clusters at the clonal and plasmid levels. In addition, the identification of a widely disseminated NDM-1 plasmid highlights its potential role in resistance spread.

## Conclusion

This study underscores the need to prioritize clonal and plasmid transmission in outbreak management of NDM-1-producing KLPN, particularly involving high-risk clones like ST147. The diversity of NDM-1 plasmids highlights their ongoing evolutionary potential and further investigations of additional NDM-1 plasmids is necessary to gain a better understanding of their occurrence and transmission dynamics. Cross-border transmission and incomplete plasmid resolution reinforce the importance of integrating long-read sequencing and international surveillance into routine resistance monitoring.

## Supplementary Information

Below is the link to the electronic supplementary material.

Supplementary Material 1

Supplementary Material 2

Supplementary Material 3

Supplementary Material 4

Supplementary Material 5

Supplementary Material 6

Supplementary Material 7

Supplementary Material 8

## Data Availability

Sequencing data are accessible in the NCBI database, BioProject ID PRJNA1250840.

## Abbreviations

cgMLST: Core genome multi locus sequence typing
Charité: Universitätsmedizin Berlin
CT: Complex type
Inc: Incompatibility group
KLPN: Klebsiella pneumoniae
MLST: Multi locus sequence typing
NCBI: National Centre for Biotechnology Information
ST: Sequence type
