# Supplementary figures and images for: NDM-1 plasmid clustering reflects clonal transmission of Klebsiella pneumoniae ST147 in four hospitals in Berlin, Germany
Source: Antimicrob Resist Infect Control. 2025 Oct 2;14:114. doi: 10.1186/s13756-025-01639-x (PMC12492617; doi:10.1186/s13756-025-01639-x)

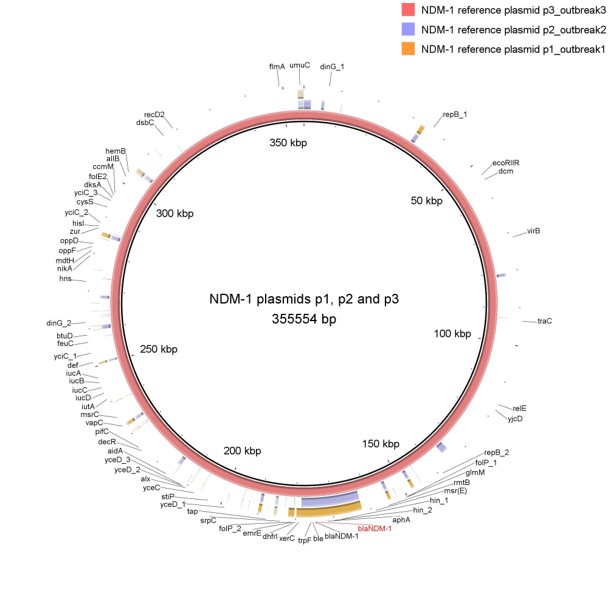

Supplement: Supplementary file 1 — Supplementary Material 1 [file 13756_2025_1639_MOESM1_ESM.jpg]
