# Supplementary material for: NDM-1 plasmid clustering reflects clonal transmission of Klebsiella pneumoniae ST147 in four hospitals in Berlin, Germany
Source: Antimicrob Resist Infect Control. 2025 Oct 2;14:114. doi: 10.1186/s13756-025-01639-x (PMC12492617; doi:10.1186/s13756-025-01639-x)
Supplement: Supplementary file 3 — Supplementary Material 3 [file 13756_2025_1639_MOESM3_ESM.pdf]

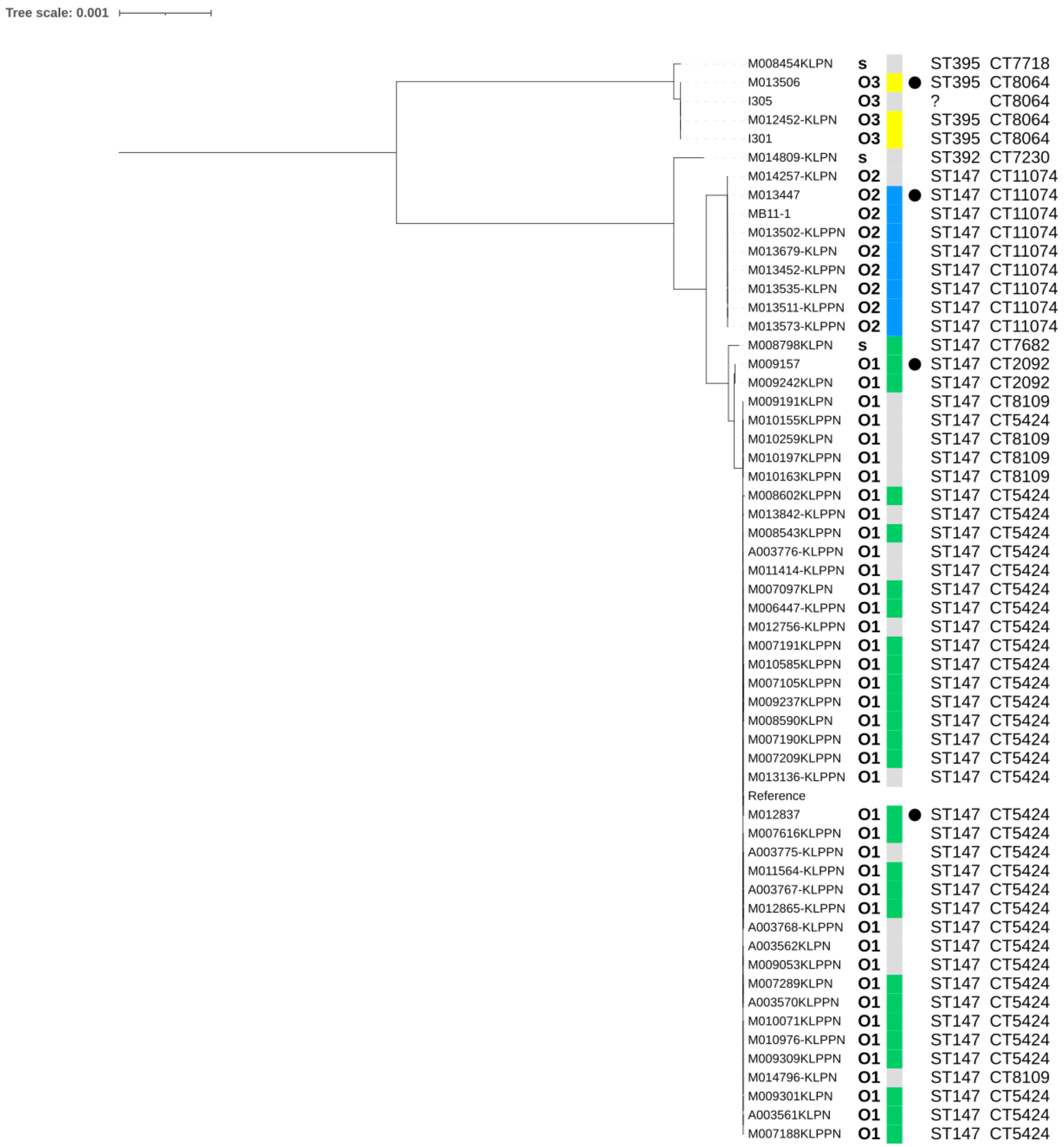

Fig S1. Maximum-likelihood phylogenetic tree of 57 *Klebsiella pneumoniae* isolates based on core genome SNP alignment. The alignment was generated using Snippy with hybrid-assembled isolate M012837 (outbreak cluster O1) as the reference. Tree construction was performed using FastTree and visualized with iTOL. (Legend: cluster outbreak assignment according cgMLST; color stripe with reference plasmid TaDReP Alignment “green” p1, “blue” p2 and “yellow” p3; long read sequenced isolates “circle”; sequence type; complex type)
